# The Effect of Astaxanthin on Fatigue in Diabetic Patients From a Nutritional Nursing Perspective

**DOI:** 10.1093/geroni/igaf122.3221

**Published:** 2025-12-31

**Authors:** Hongyu Li, Rui Zhao, Juan Li, Guifang Guo, Wei Sang

**Affiliations:** Liaoning Polytechnic Vocational University, Jinzhou, Liaoning, China (People’s Republic); Taizhou University, Faculty of Medicine, Taizhou, Zhejiang, China (People’s Republic); Liaoning Polytechnic Vocational University, Jinzhou, Liaoning, China (People’s Republic); Peking University, Beijing, Beijing, China (People’s Republic); Zhejiang Houde Mingxin Technology Service Co., Taizhou, Zhejiang, China (People’s Republic)

**Keywords:** Astaxanthin, Nutrition nursing intervention, Diabetes fatigue, Gut flora

## Abstract

Background Diabetic patients often experience increased fatigue due to inflammation, depression, and poor glycemic control. Astaxanthin, with its anti-inflammatory and antioxidant properties, has shown potential in alleviating diabetic fatigue in animal studies. Objective We aimed to investigate the effects of astaxanthin nutritional care on diabetic fatigue in a type 2 diabetes mouse model over 12 weeks, focusing on gut microbiota-related mechanisms. Methodology A type 2 diabetes mouse model was established using a 60% high-fat diet and low-dose streptozotocin injection. Mouse running exhaustion tests assessed fatigue status. Astaxanthin was added to the diet at 0.01% and 0.02% for 12 weeks. Key fatigue indicators were compared before and after intervention and between groups. Gut microbiota was analyzed via 16S rRNA sequencing. Results The diabetes model group showed significantly higher fasting blood glucose (p < 0.01), insulin (p = 0.009), and insulin resistance index (p = 0.002) than controls. Astaxanthin significantly reduced serum LPS (by 22.70% and 29.88%) and TMAO (by 33.82% and 28.57%). Gut microbiota diversity was modulated, with increased beneficial genera and suppressed harmful genera, influencing fatigue and renal function. Discussion The study demonstrated that increasing beneficial gut genera and inhibiting harmful genera effectively alleviated diabetic fatigue. Future research should explore long-term effects and human applications.

